# Definition of the Post-COVID syndrome using a symptom-based Post-COVID score in a prospective, multi-center, cross-sectoral cohort of the German National Pandemic Cohort Network (NAPKON)

**DOI:** 10.1007/s15010-024-02226-9

**Published:** 2024-04-08

**Authors:** Katharina S. Appel, Carolin Nürnberger, Thomas Bahmer, Christian Förster, Maria Cristina Polidori, Mirjam Kohls, Tanja Kraus, Nora Hettich-Damm, Julia Petersen, Sabine Blaschke, Isabel Bröhl, Jana Butzmann, Hiwa Dashti, Jürgen Deckert, Michael Dreher, Karin Fiedler, Carsten Finke, Ramsia Geisler, Frank Hanses, Sina M. Pütz, Björn-Erik O. Jensen, Margarethe Konik, Kristin Lehnert, Susana M. Nunes de Miranda, Lazar Mitrov, Olga Miljukov, Jens-Peter Reese, Gernot Rohde, Margarete Scherer, Kristin Tausche, Johannes J. Tebbe, Jörg Janne Vehreschild, Florian Voit, Patricia Wagner, Martin Weigl, Christina Lemhöfer

**Affiliations:** 1 Center for Internal Medicine, Medical Department 2 (Hematology/Oncology and Infectious Diseases), Goethe University Frankfurt, University Hospital, Theodor-Stern-Kai 7, 60596 Frankfurt am Main, Germany; 2 Department I of Internal Medicine, University of Cologne, Faculty of Medicine and University Hospital Cologne, Cologne, Germany; 3 Institute for Clinical Epidemiology and Biometry, University of Würzburg, Würzburg, Germany; 4 Institute for Medical Data Science, University Hospital Würzburg, Würzburg, Germany; 5 Internal Medicine Department I, University Hospital Schleswig-Holstein Campus Kiel, Kiel, Germany; 6 Airway Research Center North (ARCN), German Center for Lung Research (DZL), Grosshansdorf, Germany; 7 Institute of General Practice and Interprofessional Care, University Hospital Tübingen, Tübingen, Germany; 8 Department II of Internal Medicine and Center for Molecular Medicine Cologne, University of Cologne, Faculty of Medicine and University Hospital Cologne, Cologne, Germany; 9 CECAD, University of Cologne, Faculty of Medicine and University Hospital Cologne, Cologne, Germany; 10 Department of Psychosomatic Medicine and Psychotherapy, University Medical Center Mainz, Mainz, Germany; 11 Emergency Department, University Medical Center Göttingen, Göttingen, Germany; 12 Medical Faculty, Institute of Medical Microbiology and Hospital Hygiene, University Hospital Magdeburg, Otto-Von-Guericke University Magdeburg, Magdeburg, Germany; 13 Practice for General Medicine Dashti, Eberswalde, Germany; 14 Center of Mental Health, Department of Psychiatry, Psychosomatics and Psychotherapy, University Hospital Würzburg, Würzburg, Germany; 15 Department of Pneumology and Intensive Care Medicine, University Hospital RWTH Aachen, Aachen, Germany; 16 Department of Neurology, Charité Berlin, Berlin, Germany; 17 Emergency Department and Department for Infection Control an Infectious Diseases, University Hospital Regensburg, Regensburg, Germany; 18 Department I of Internal Medicine, Center for Integrated Oncology Aachen Bonn Cologne Duesseldorf, University of Cologne, Faculty of Medicine and University Hospital Cologne, Cologne, Germany; 19 Department of Gastroenterology, Hepatology and Infectious Diseases, Medical Faculty, Düsseldorf University Hospital, Heinrich Heine University, Düsseldorf, Germany; 20 Department of Infectious Diseases, West German Centre of Infectious Diseases, University Medicine Essen, University Duisburg-Essen, Essen, Germany; 21 DZHK (German Center for Cardiovascular Research), University Medicine Greifswald, Greifswald, Germany; 22 Department of Internal Medicine B, University Medicine Greifswald, Greifswald, Germany; 23 Department of Respiratory Medicine, Goethe University Frankfurt, University Hospital, Medical Clinic I, Frankfurt/Main, Germany; 24 Department of Internal Medicine I, University Hospital Carl Gustav Carus TU Dresden, Dresden, Germany; 25 Department of Gastroenterology and Infectious Diseases, Klinikum Lippe, Lippe, Germany; 26 German Centre for Infection Research (DZIF), Partner Site Bonn-Cologne, Cologne, Germany; 27 Department of Internal Medicine II, School of Medicine, University Hospital Rechts der Isar, Technical University of Munich, Munich, Germany; 28 Department of Orthopaedics and Trauma Surgery, Musculoskeletal University Center Munich (MUM), University Hospital, LMU Munich, Munich, Germany; 29 Institute of Physical and Rehabilitation Medicine, Jena University Hospital/Friedrich-Schiller-University Jena, Jena, Germany

**Keywords:** Post-COVID, Long-COVID, Definition, Score, Patient reported outcome measures, Multi-center prospective cohort study, Quality of life

## Abstract

**Purpose:**

The objective examination of the Post-COVID syndrome (PCS) remains difficult due to heterogeneous definitions and clinical phenotypes. The aim of the study was to verify the functionality and correlates of a recently developed PCS score.

**Methods:**

The PCS score was applied to the prospective, multi-center cross-sectoral cohort (in- and outpatients with SARS-CoV-2 infection) of the "National Pandemic Cohort Network (NAPKON, Germany)". Symptom assessment and patient-reported outcome measure questionnaires were analyzed at 3 and 12 months (3/12MFU) after diagnosis. Scores indicative of PCS severity were compared and correlated to demographic and clinical characteristics as well as quality of life (QoL, EQ-5D-5L).

**Results:**

Six hundred three patients (mean 54.0 years, 60.6% male, 82.0% hospitalized) were included. Among those, 35.7% (215) had no and 64.3% (388) had mild, moderate, or severe PCS. PCS severity groups differed considering sex and pre-existing respiratory diseases. 3MFU PCS worsened with clinical severity of acute infection (*p* = .011), and number of comorbidities (*p* = .004). PCS severity was associated with poor QoL at the 3MFU and 12MFU (*p* < .001).

**Conclusion:**

The PCS score correlated with patients’ QoL and demonstrated to be instructive for clinical characterization and stratification across health care settings. Further studies should critically address the high prevalence, clinical relevance, and the role of comorbidities.

**Trail registration number:**

The cohort is registered at www.clinicaltrials.gov under NCT04768998.

**Supplementary Information:**

The online version contains supplementary material available at 10.1007/s15010-024-02226-9.

## Background

Three years after the 2019 coronavirus (COVID-19) pandemic, the long-term consequences of COVID-19 in terms of prognosis, quality of life and ability to work have become the focus of medical research [1, 2]. The World Health Organization (WHO) has defined the Post-COVID syndrome (PCS), also known as Long-COVID or Post-COVID condition (PCC), as a condition that may affects patients after severe acute respiratory syndrome coronavirus type 2 (SARS-CoV-2) infection and results in symptoms that appear or persist three months after diagnosis, last for at least two months, and cannot be explained by any other diagnosis [3]. Other definitions also exist, e.g., from the UK National Institute for Health and Care Excellence (NICE) [4] and the Centers for Disease Control and Prevention (CDC) [5]. As most PCS studies do not adhere to one of these definitions, they contribute to further heterogeneity by using a diverse set of symptoms and time points [6, 7]. Given diverse diagnostic criteria and clinical phenotypes, there is a significant lack of objective and standardized measures to investigate the pathogenesis, prevalence, and effective treatment of PCS.

To complicate matters, there are difficulties in applying these broad criteria to existing cohort studies designed in the early phase of the pandemic, when PCS was neither widely known nor well defined among researchers. Currently, researchers are searching for existing data suggesting PCS based on different study designs (follow-up time points, duration of symptoms) and using symptom clusters of varying magnitude in their respective cohorts [6–9]. In addition, it is difficult, but important, to disentangle the causal impact of the COVID-19 infection from other pandemic-related illnesses (e.g. psychiatric disorders due to social isolation) [10, 11] or pre-existing comorbidities [12]. This need is emphasized by the WHO, which limits itself to symptoms for which there is "no other explanation" [3]. The diversity of symptoms in PCS raises the question of whether there are distinct clinical phenotypes [13]. Researchers propose different clinical profiles, symptom clusters, or scoring systems [13–16], leading to challenges in comparability and assessing PCS quantification and clinical relevance.

At the beginning of the COVID-19 pandemic, Germany established the “Network of University Medicine (NUM)” to coordinate and support national research on COVID-19. As part of this effort, the “National Pandemic Cohort Network (NAPKON)” was created. NAPKON is a multi-center prospective observational cohort study consisting of three cohorts with comprehensive long-term follow-up schemes [17]. With the shift in research priorities towards PCS, the NAPKON cohorts require a standardized, easily applicable PCS definition to further investigate the pathogenesis of PCS. Bahmer et al. (2022) developed the Post-COVID syndrome score (PCS score) based on the data of the population-based cohort in NAPKON (“Populationsbasierte Platform”, POP), providing a useful tool for evaluating the presence and severity of PCS symptoms [14].

## Objective

Our aim was to implement the PCS score developed by Bahmer et al. in the cross-sectoral cohort (“Sektorenübergreifende Plattform”, SUEP) of NAPKON and to help standardize and objectify the definition of symptom-based PCS across the network. To achieve this, we calculated the symptom complexes-based PCS score using two approaches: firstly, we mapped the symptom complexes only to patient information obtained during follow-up visits at 3 and 12 months after initial infection. Secondly, we utilized Patient Reported Outcome Measures (PROMs) to identify the occurrence of individual symptoms. We analyzed the frequency of the PCS score severity groups stratified by relevant demographic, disease-related information and risk factors and compared PCS score severity with the QoL outcomes. Additionally, we sought to obtain an initial assessment of the relationship between the PCS score and functional and social impairments and evaluated changes in the PCS score over time. These results are critically discussed in the context of other PCS definitions/scores and relevant challenges in the field.

## Methods

### Study design and sample

The SUEP cohort of NAPKON recruited SARS-CoV-2 positive tested patients and controls of all ages in all sectors of health care (university hospitals, non-university hospitals, and primary care practices) in Germany, encompassing in- and outpatients since November 4, 2020. The SUEP collects primary health record data, basic clinical phenotyping information, imaging data, bio samples, and PROMs at 57 study sites. The key inclusion criterion was a positive polymerase chain reaction (PCR)-confirmed SARS CoV-2 infection at the time of inclusion. Among other visits, the patients received either telephone or on-site follow-up examinations at three and 12 months (referred to as 3MFU and 12MFU, respectively) after their initial baseline visit. The choice between telephone and on-site follow-ups depended on the heath care level or consent to bio sample collection. Detailed information about the study was published elsewhere [17]. Only adults (age ≥ 18 years) who had at least a 3MFU were eligible for our analysis. A complete flow chart depicting sample selection and dropouts during analysis is shown in Supplementary Fig. S1. Our sample was recruited between December 2, 2020 and October 25, 2022. The last included 12MFU was carried out on May 31, 2023. Data were exported on June 15, 2023. All reporting adheres to the STROBE guidelines for cohort studies (Supplementary File S1).

### Post-COVID syndrome score (PCS score) mapping and enhancement

The PCS score was calculated based on self-reported symptoms using a hypothesis-free clustering procedure that employed k-means clustering alongside other techniques. It consists of 12 subordinate sets of symptoms, including chemosensory deficits, fatigue, exercise intolerance, joint or muscle pain, ear-nose-throat ailments, coughing/wheezing, chest pain, gastrointestinal ailments, neurological ailments, dermatological ailments, infection signs, and sleep disturbance, as reported by Bahmer et al. [14]. If a symptom of the respective symptom complexes was present, the symptom indicators were multiplied by an individual point value ranging from two to seven representing its contribution to PCS severity. The assigned points were then summed up to a total score, which can be used to categorize the severity classes of PCS into distinct classes (none: 0 points; mild: > 0 to ≤ 10.75; moderate > 10.75 to ≤ 26.25; severe: > 26.25).

For mapping the PCS score in the SUEP, we identified 25 symptom items suitable to represent the 12 complexes. A list of included data items can be found in Table S1. In the SUEP, symptoms were assessed continuously and longitudinally during the course of the study. Symptom occurrence was further specified by start date and duration using categorical time intervals (e.g.“1 day”,“8–14 days”, “31–60 days (1–2 months)”). In the following analyses, we refer to the 3MFU, unless otherwise specified.

In order to meet the WHO definition of Post-COVID, symptoms must have persisted for at least two months prior to 3MFU [3]. Therefore, we calculated the time intervals between the onset of symptom and the visit date. We used the categorical duration intervals to determine a minimum and a maximum end date. A symptom complex was considered present if the start date was at least 61 days prior to the 3MFU date and the minimum end date was not earlier than the 3MFU date. To evaluate changes in the PCS score severity over time, we also calculated the PCS score during the 12MFU visit. In order to establish a clearer link between symptoms and infection, it was necessary for symptoms to persist for at least 11 months (335 days) and the “6–12 months “ duration category had to be selected. For symptoms labeled as “ongoing”, we used the date on which the corresponding template was last updated as the symptom end date.

After conducting the initial analysis, we discovered that some symptom complexes, e.g. fatigue, were underrepresented in comparison to existing literature [14, 18, 19]. To address this, we included additional elements from the following PROMs in our assessment: Chalder Fatigue Scale (CFS), PROMIS-29 Fatigue, PROMIS Dyspnea, PROMIS Cognitive Function, and PROMIS-29 Sleep disturbance (see Table S1). As suggested by Jackson [20], we recoded the CFS items to dichotomous items, created a sum score and categorized it as either “no fatigue” (sum score ≤ 3) or “fatigue” (sum score > 3). For PROMIS-29 Fatigue, PROMIS Dyspnea, PROMIS Cognitive Function and PROMIS-29 Sleep disturbance, we calculated sum scores and categorized these using T-Scores. T-Scores < 55 indicate no impairments for fatigue, dyspnea and sleep, while T-scores ≥ 55 indicate impairments, respectively [21]. The corresponding sum scores for fatigue [22], dyspnea [23], and sleep [24] were 11, 15, and 13, respectively. Cognitive impairments were indicated by a T-score ≤ 45, while a T-score ≥ 45 indicated no cognitive impairments [25]. The corresponding sum score was 15 [21]. If one item was missing or answered as “no information available”, a sum score could not be calculated for PROMIS-29 Fatigue, PROMIS Cognitive Function and PROMIS-29 Sleep Disturbance. To calculate a sum score for PROMIS Dyspnea, at least four items needed to be answered. Supplementary Text 1 details versions of the PROMs used in the SUEP. In the following analyses, we primarily used the mapping version that includes the PROM assessments, aligning with the fatigue prevalence reported in the literature [9, 13].

If a PROM sum score could not be calculated or the start date or duration of all symptoms per symptom complex was missing, the corresponding complex was marked as missing. If at least one complex was missing, no score was calculated.

### Assessment of functional impairments and quality of life

To identify the effects and classify clinical relevance of PCS severity on physical and social impairments, we evaluated the PROMIS-29 “Ability to participate in social roles and activities” and “Physical function”. We categorized impairment and no impairment using a sum score cut-off of 13 and 18, respectively [26, 27]. QoL was measured using the European Quality of Life 5-Dimensions 5-Level Version (EQ-5D-5L) index and Visual Analogue Scale (VAS). The study assessed the health status of patients based on five dimensions: mobility, caring for oneself, everyday activities, pain, and anxiety. The patients rated each dimension on a five-point Likert-scale ranging from 1 (no problems) to 5 (inability/extreme problems). The scores were then calculated into an index score between 0 (worst condition) and 1 (optimal health) [28, 29]. Additionally, the patients reported their present health status on the VAS with 0 representing the worst health status and 100 the best health status.

### Data analysis

Mean (M) and standard deviation (SD) were calculated for metric items, and absolute frequencies and percentages were reported for categorical and dichotomous items. Significance of group differences was assessed using the Chi-squared test or Fisher‘s exact test for categorical items, as appropriate. Due to the lack of a normal distribution for all continuous items (Shapiro–Wilk-Test), group differences were assessed using the Kruskal–Wallis-Test. Post-hoc tests were performed using Dunn’s-test. When appropriate, *p* values were Bonferroni-adjusted. Correlation analyses were performed using Spearman-correlation, with Spearman’s correlation coefficient *rho* considered to be small if |*rho*| > *0.1*, medium if |*rho*| > *0.3* and large if |*rho*| > *0.5* [30]. All *p* values below *0.05* were considered statistically significant. We performed sensitivity analysis by treating missing symptom clusters as “not present”. R Version 4.2.3 was used for statistical analysis.

## Results

### Patient characteristics

The study sample comprised 854 patients, all of whom completed the 3MFU, from 27 study centers. The calculation of PCS score was possible for 581 patients (68.0%) without PROMs and for 603 patients (70.6%) with PROMs at 3MFU. Of those patients with PCS score including PROMs, 97.0% had a 12MFU (*n* = 585). The mean age of those patients with valid PCS score (including PROMs) was 54.0 years (SD = 16.1). Approximately 61% of patients (*n* = 365) were male. During the acute phase of COVID-19 infection, 81.6% of patients (*n* = 492) were hospitalized. Of these, 82.7% (*n* = 407) did not receive oxygen or received oxygen with less than 15 l/min (equal to WHO progression scale moderate). Forty-three percent of patients received at least one vaccination against SARS-CoV-2 prior to study inclusion (n = 240), of whom 45.4% (*n* = 179) received all reported vaccinations prior to study inclusion. Of the patients, 28.0% (*n* = 157) received two vaccinations, while 26.1% (*n* = 146) received three or more doses. The most frequently mentioned pre-existing comorbidities were cardiovascular diseases (*n* = 278, 46.1%), diabetes (*n* = 101, 16.8%), and cancer (*n* = 103, 17.1%). Twenty-nine percent had no comorbidities, while 70.7% of patients had at least one comorbidity. Please refer to Table 1 for further information.

**Table 1:** Cohort characteristics and group comparisons between PCS score groups at 3MFU

	Total		PCS score^a^								*p* value^b^	
			0 (none)		> 0 to ≤ 10.75 (mild)		> 10.75 to ≤ 26.25 (moderate)		> 26.25 (severe)		Unadjusted	Adjusted
n (%)	603	(100.0)	215	(35.7)	120	(19.9)	221	(36.7)	47	(7.8)		
Age [years]*, mean (SD)*^c,d^	54.0	(16.1)	52.0	(17.1)	54.9	(17.3)	55.3	(14.9)	54.7	(12.1)	0.207	n.a
Age groups, *n (%)*^c,d^												
< 65 years	438	(72.9)	155	(72.1)	82	(68.9)	163	(74.1)	38	(80.9)	0.442	n.a
≥ 65 years	163	(27.1)	60	(27.9)	37	(31.1)	57	(25.9)	9	(19.1)		
Sex* n (%)*^c,d^												
Women	237	(39.4)	70	(32.6)	48	(40.3)	91	(41.2)	28	(59.6)	0.006*	0.034*
Men	365	(60.6)	145	(67.4)	71	(59.7)	130	(58.8)	19	(40.4)		
BMI [kg/m^2^], *mean (SD)*^c,d^	27.9	(6.1)	27.5	(6.0)	27.2	(6.0)	28.4	(6.1)	28.9	(5.9)	0.077	n.a
Smoker, *n (%)*^d^												
Never	349	(59.1)	126	(61.2)	69	(58.0)	127	(58.0)	27	(57.4)	0.899	n.a
Yes or former	242	(40.9)	80	(38.8)	50	(42.0)	92	(42.0)	20	(42.6)		
Clinical Severity (following WHO criteria)*, n (%)*^c,e^												
Mild disease (no hospitalization)	108	(18.0)	50	(23.3)	25	(21.0)	27	(12.3)	6	(12.8)	0.038*	0.687
Moderate disease (hospitalized, no oxygen or oxygen by mask or nasal prongs (< 15 l/min))	407	(67.8)	140	(65.1)	73	(61.3)	158	(72.1)	36	(76.6)		
Severe disease (hospitalized, oxygen by NIV or high flow (> 15 l/min))	85	(14.2)	25	(11.6)	21	(17.6)	34	(15.5)	5	(10.6)		
Hospitalized, *n (%)*^c,e^												
No	108	(18.0)	50	(23.3)	25	(21.0)	27	(12.3)	6	(12.8)	0.015*	0.093
Yes	492	(82.0)	165	(76.7)	94	(79.0)	192	(87.7)	41	(87.2)		
Admission to intensive care unit (ICU)*, n (%)* ^c,e^												
Never	398	(83.3)	140	(84.8)	76	(80.9)	159	(82.8)	37	(90.2)	0.514	n.a
At least once	80	(16.7)	25	(15.2)	18	(19.1)	33	(17.2)	4	(9.8)		
At least one vaccination against Sars-COV-2 prior to study inclusion*, n (%)*^c,h^												
No	313	(56.6)	101	(51.8)	67	(61.5)	117	(56.8)	28	(65.1)	0.245	n.a
Yes	240	(43.4)	94	(48.2)	42	(38.5)	89	(43.2)	15	(34.9)		
All reported vaccination against SARS-CoV-2 prior to study inclusion, *n (%)*^*b,c*^												
No	215	(54.6)	60	(45.1)	49	(62.8)	96	(60.4)	10	(41.7)	0.013*	0.080
Yes	179	(45.4)	73	(54.9)	29	(37.2)	63	(39.6)	14	(58.3)		
No.of vaccinations per patient, *mean (SD)*^*c, i*^	1.6	(1.3)	2.2	(0.9)	2.2	(1.0)	2.2	(0.9)	2.3	(1.1)	0.977	n.a
0, *n (%)*	159	(28.4)	62	(31.3)	31	(27.9)	47	(22.6)	19	(44.2)	0.224	n.a
1, *n (%)*	98	(17.5)	33	(16.7)	23	(20.7)	35	(16.8)	7	(16.3)		
2, *n (%)*	157	(28.0)	53	(26.8)	28	(25.2)	68	(32.7)	8	(18.6)		
3 or more, *n (%)*	146	(26.1)	50	(25.3)	29	(26.1)	58	(27.9)	9	(20.9)		
Quality of life (EQ-5D-5L) at 3MFU, *mean (SD)*^c^												
Index (0–1)	0.8	(0.2)	0.9	(0.2)	0.9	(0.2)	0.8	(0.2)	0.7	(0.2)	< 0.001*	< 0.001*
VAS (0–100 points)	73.9	(20.3)	86.4	(13.7)	75.8	(20.5)	65.8	(19.7)	60.3	(17.1)	< 0.001*	< 0.001*
No. of pre-existing comorbidities*, mean (SD)*^j,k,l^	1.9	(1.9)	1.5	(1.6)	2.1	(2.1)	2.0	(2.0)	2.1	(2.0)	0.016*	0.098
0, *n (%)*	177	(29.4)	74	(34.4)	35	(29.2)	56	(25.3)	12	(25.5)	0.194	n.a
1 or more,* n (%)*	426	(70.7)	141	(65.6)	85	(70.8)	165	(74.7)	35	(74.5)		
1–2,* n (%)*	252	(41.8)	92	(42.8)	45	(37.5)	93	(42.1)	22	(46.8)		
3–5,* n (%)*	138	(22.9)	43	(20.0)	28	(23.3)	58	(26.2)	9	(19.1)		
6 or more,* n (%)*	36	(6.0)	6	(2.8)	12	(10.0)	14	(6.3)	4	(8.5)		
Pre-existing comorbidities* n (%)*^c,j,k,l^												
Cardiovascular diseases	278	(46.1)	94	(43.7)	62	(51.7)	103	(46.6)	19	(40.4)	0.454	n.a
Diabetes	101	(16.8)	29	(13.6)	29	(24.2)	34	(15.5)	9	(19.1)	0.081	n.a
Cancer	103	(17.1)	35	(16.3)	16	(13.3)	41	(18.6)	11	(23.4)	0.394	n.a
Allergies	90	(15.0)	29	(13.6)	21	(17.5)	34	(15.4)	6	(13.0)	0.772	n.a
Respiratory diseases	74	(12.3)	10	(4.7)	14	(11.7)	42	(19.0)	8	(17.0)	< 0.001*	< 0.001*
Nephrological diseases	64	(10.6)	19	(8.8)	14	(11.7)	23	(10.4)	8	(17.0)	0.383	n.a
Gastrointestinal or hepatic diseases	40	(6.6)	11	(5.1)	8	(6.7)	18	(8.1)	3	(6.4)	0.654	n.a
Organ transplant	35	(5.8)	14	(6.5)	8	(6.7)	10	(4.5)	3	(6.4)	0.734	n.a
Psychiatric diseases	35	(5.8)	7	(3.3)	3	(2.5)	21	(9.5)	4	(8.7)	0.009*	0.056
Neurological diseases	23	(3.8)	7	(3.3)	5	(4.2)	7	(3.2)	4	(8.5)	0.330	n.a
Rheumatologic or immunologic diseases	13	(2.2)	1	(0.5)	2	(1.7)	8	(3.6)	2	(4.3)	0.058	n.a
PROMIS-29 Ability to participate in social roles and activities, *mean (SD)*^c,d^	15.4	(4.6)	19.0	(2.0)	16.5	(4.1)	13.2	(4.4)	11.7	(3.8)	< 0.001*	< 0.001*
No social impairments*, n (%)*	297	(71.6)	113	(98.3)	74	(87.1)	96	(54.5)	14	(35.9)	< 0.001*	< 0.001*
Social impairments*, n (%)*	118	(28.4)	2	(1.7)	11	(12.9)	80	(45.5)	25	(64.1)		
PROMIS-29 Physical function*, mean (SD)* ^c,d^	16.9	(4.0)	19.4	(1.5)	17.4	(3.7)	15.5	(4.3)	14.2	(4.2)	< 0.001*	< 0.001*
No physical impairments*, n (%)*	253	(60.0)	111	(93.3)	57	(64.8)	73	(41.2)	12	(31.6)	< 0.001*	< 0.001*
Physical impairments*, n (%)*	169	(40.0)	8	(6.7)	31	(35.2)	104	(58.8)	26	(68.4)		

^a^PCS-Groups including PROMs, missing values 251

^b^group differences assessed by Chi2, Fishers- or Kruskal–Wallis test, as appropriate; normally significant values were Bonferroni-adjusted by multiplication with the size of the respective group characteristics (i.e. 6 or 18), n.a: not applicable because the unadjusted p value already exceeded 0.050

^c^all analyses were conducted for patients with available data and calculated PCS score (missing data: age/age groups 2, sex: 1, BMI 52, smoker 12, clinical severity 3, hospitalization 5, admission to ICU 14, vaccination against SARS-CoV-2 prior to study inclusion 50, number of vaccinations 43,EQ-5D-5L Index (3MFU) 73, EQ-5D-5L VAS (3MFU) 88, PROMIS-29 Ability to participate in social roles and activities 188, PROMIS-29 Physical function 188, diabetes 2, cancer 1, allergies 3, psychiatric diseases 1)

^d^for post-hoc test results see Table S2 (age, BMI, number of comorbidities, PROMIS-29 Ability to participate in social roles and activities, PROMIS-29 Physical function), Table S3 (age groups (< or ≥65 years), sex, smoker, hospitalization, administration to ICU), Table S4 (clinical severity)

^e^during acute phase (baseline to end acute phase) of SARS-CoV-2 infection

^f^of those patients who had been hospitalized at least once during acute phase

^g^“worst” care unit per patient

^h^“No” includes all patients without any SARS-CoV-2 vaccination; “Yes” includes all patients with at least one SARS-CoV-2 vaccination prior to study visit

^i^only patients with vaccination against SARS-CoV-2 included; “all reported vaccinations against SARS-CoV-2 prior to study inclusion” means that the time point of all vaccinations that were reported by patients, lay prior to the inclusion to the study, irrespective whether the patients had a “full vaccination” at the time of study inclusion; “full vaccination” depends on the point in time, the availability and the guideline recommendations during the course of the pandemic (e.g. full vaccination after two doses or only after third “booster” vaccination); “no. of vaccinations against SARS-CoV-2” refers to the total amount of doses a patient received irrespective of the point of time the patient was vaccinated (e.g. a patient had three vaccinations in total but might have had only two prior to study inclusion which would lead to him/her not being included in “Yes” in “all reported vaccinations against SARS-CoV-2 prior to study inclusion”)

^j^all information on pre-existing comorbidities was extracted from physician's letters. ‘Pre-existing’ refers to the time before SARS-CoV-2 infection. Date of diagnosis was compared to baseline date. The total list of comorbidities underlying the corresponding categorization derived from the German Corona Consensus Dataset (GECCO-83), the common core data set of the NAPKON project

^k^at least one of the diseases was diagnosed prior to baseline visit (respiratory diseases: asthma, COPD, pulmonary fibrosis, pulmonary hypertension, obesity hypoventilation syndrome, sleep apnea, obstructive sleep apnea syndrome, cystic fibrosis; cardiovascular diseases: hypertension, CHD, heart attack, balloon dilatation/revascularization, heart failure chronic or acute, peripheral occlusive disease (pAVK), carotid stenosis, atrial fibrillation; neurological diseases: Parkinson, dementia, multiple sclerosis, epilepsy, migraine, stroke hemorrhagic or ischemic; psychiatric diseases: psychosis, depression, anxiety disorder; gastrointestinal and hepatic diseases: gastric ulcer, chronic inflammatory bowel disease, acute pancreatitis, fatty liver, alcoholic liver disease, cirrhosis of the liver, chronic infectious hepatitis B or C, autoimmune liver disease; rheumatologic or immunologic diseases: rheumatoid arthritis, collagenases, vasculitis, congenital immunodeficiency; nephrological diseases: chronic kidney disease; allergies: penicillin, pollen, dust, mold; cancer: hematooncological disease, solid tumor disease; organ transplant: heart, liver, lung, pancreas, kidney)

^l^pre-existing comorbidities were either diagnosed or not diagnosed

### Symptom complexes and Post-COVID syndrome score (PCS score)

**Fig. 1:**
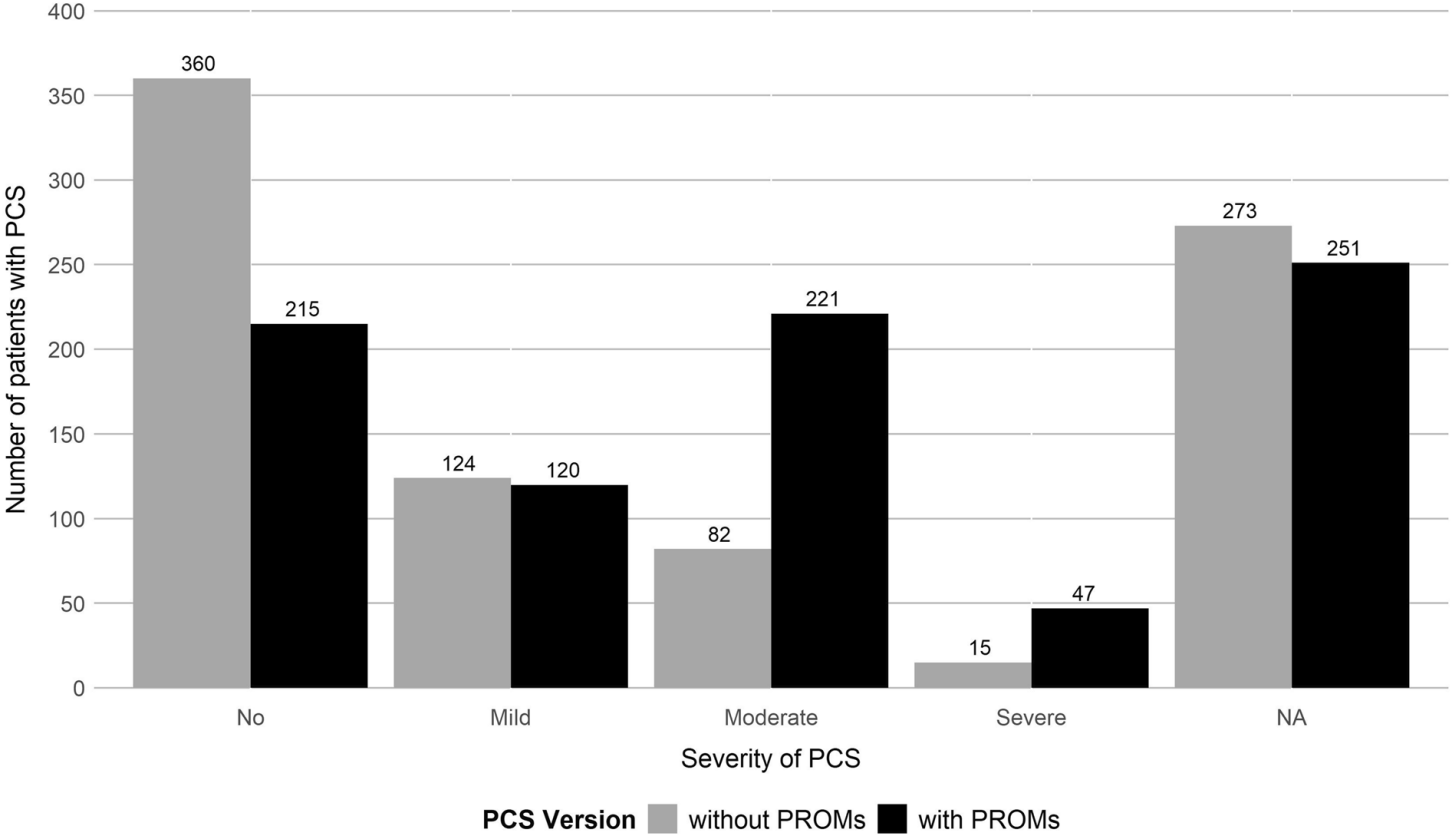
Presence of PCS at the 3MFU according to the PCS score by Bahmer et al. [14] with or without Patient Reported Outcome Measures (PROMs)

For PCS score mapping, prior to PROM inclusion, exercise intolerance as demonstrated by shortness of breath was the most frequently reported symptom complex (20.3%), followed by neurological ailments (12.2%) and coughing/wheezing (10.4%). None of the patients reported sleep disturbances (in the free text field). After PROM inclusion, fatigue was most common (41.1%), followed by neurological ailments (39.4%) and exercise intolerance (39.7%). Sleep disturbances according to PROMs were present in 140 patients (16.4%). Detailed information is given in Table 2. The PCS score group allocation shifted from 62.0% (*n* = 360) to 35.7% (*n* = 215) for patients with no, 14.5% (*n* = 124) to 14.1% (*n* = 120) with mild, 14.1% (*n* = 82) to 36.7% (*n* = 221) with moderate and 2.6% (*n* = 15) to 7.8% (*n* = 47) with severe PCS after PROM integration (Fig. 1).

**Table 2:** Symptom complexes with symptoms present for at least two months at the 3MFU visit as recommended by Bahmer et al. [14]

No	Symptom complex	Self-reported sub-symptoms	Without PROMs		With PROMs	
			*n*	(%)	*n*	(%)
1	Chemosensory deficits^a,b^	Smelling disturbance, impaired sense of taste	73	(9.4)	73	(9.4)
2	Fatigue^b^	Fatigue	68	(8.3)	341	(41.1)
3	Exercise intolerance	Shortness of breath	150	(20.3)	307	(39.7)
4	Joint or muscle pain^a,b^	Muscle pain, joint pain	60	(7.7)	60	(7.7)
5	Ear-Nose-Throat (ENT) ailments^a,b^	Sneezing, sore throat, running nose, stuffy nose	37	(4.7)	37	(4.7)
6	Coughing, wheezing^a,b^	Coughing, wheezing	82	(10.4)	82	(10.4)
7	Chest pain^b^	Chest pain	21	(2.8)	79	(10.2)
8	Gastrointestinal ailments^b^	Stomach pain, diarrhea, vomiting, nausea	38	(4.6)	74	(9.0)
9	Neurological ailments^b^	Confusion, vertigo, headache, deficits of cognition	98	(12.2)	321	(39.4)
10	Dermatological ailments^a,b^	Skin or mucous membrane change	12	(1.6)	12	(1.6)
11	Infection signs^a,b^	Chills, fever, feeling ill, lymph node swelling, loss of appetite	39	(4.7)	39	(4.7)
12	Sleep disturbance^b^	Sleep disturbance, not elsewhere classified	0	(0.0)	140	(16.4)

^a^no adjustment with patient reported outcome measures (PROMs)

^b^the percentages presented relate to the number of patients with available data (missing data: chemosensory deficits 80, Fatigue without/with PROMs 31/25, exercise intolerance without/with PROMs 114/81, joint or muscle pain 79, ENT ailments 72, coughing/wheezing 62, chest pain without/with PROMs 91/81, gastrointestinal ailments without/with PROMs 29/29, neurological ailments without/with PROMs 54/39, dermatological ailments 82, infection signs 28, sleep disturbance without/with PROMs 0/0)

### Differences between PCS score groups

When comparing PCS score groups, we found higher frequencies for female sex (*p* = 0.034*) and the presence of pre-existing respiratory diseases (*p* < 0.001*) (Table 1). Post-hoc tests showed that the comparisons between sex groups were only significant between no vs. severe (*p* < 0.001*), mild vs. severe (*p* = 0.039*) and moderate vs. severe PCS (*p* = 0.032*). QoL (measured using EQ-5D-5L index and VAS) differed significantly between PCS score groups at 3MFU and 12MFU (all p < 0.001*). Tables S2 to S4 display additional group comparisons and post-hoc tests. Stratified by sex and age, significant differences were observed between hospitalization and PCS score groups for female patients under 65 years of age (*p* = 0.035*) (Table S6). Post-hoc tests revealed significant differences between no vs. moderate (*p* = 0.013*), no vs. severe (*p* = 0.020*), and mild vs. severe PCS (*p* = 0.048*) (Table S7).

Correlation analyses showed a small but significant correlation between the PCS score and increasing BMI (*p* = 0.004*, *rho* = 0.121). This correlation was only present for females younger than 65 years (*p* = 0.001*, *rho* = 0.249) when stratified by sex and age. Similar effects were found for clinical severity at acute infection in general (*p* = 0.011*, *rho* = 0.104) and when stratified for female patients younger than 65 years (*p* = 0.002*, *rho* = 0.226). The number of pre-existing comorbidities increased as PCS score increased (*p* = 0.004*), but the effect size was small (*rho* = 0.116). When stratified by sex and age, the correlation was only significant for male patients (< 65 years: *p* = 0.006*, *rho* = 0.170; ≥ 65 years: *p* = 0.018*, *rho* = 0.229). The EQ-5D-5L index and VAS correlated significantly with increasing PCS score values at 3 and 12MFU (all p < 0.001*). This correlation had a strong negative effect (all rho ≤ -0.5) indicating a decreasing QoL with increasing PCS score (Table S8).

### Changes in PCS severity from 3 to 12 month follow-up

**Fig. 2:**
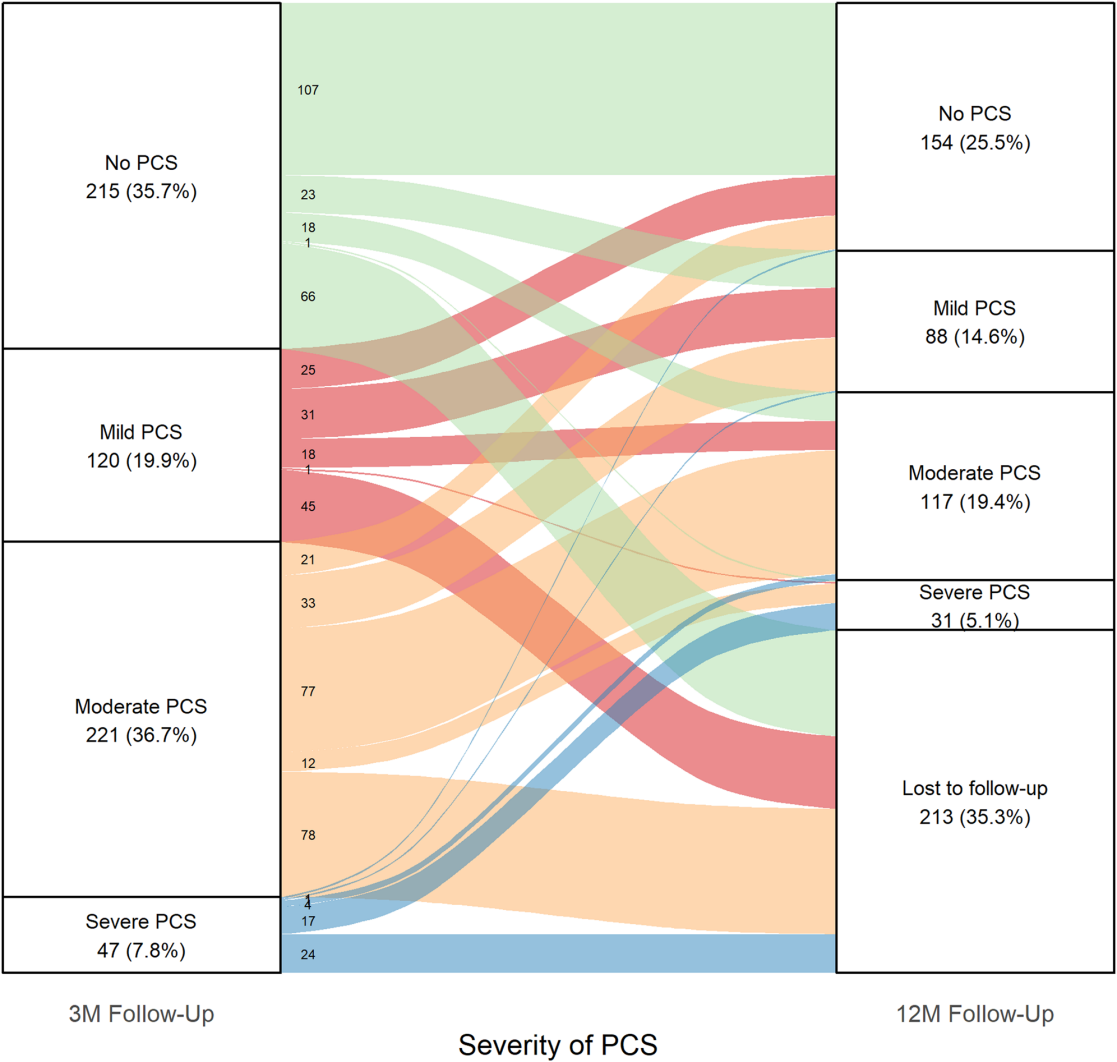
Changes in the severity of PCS scores between the 3MFU and the 12MFU

Of the 603 patients who had a PCS score at 3MFU, 68.5% (*n* = 413) also had a PCS score at 12MFU. Among them, 12.1% experienced a worsening in PCS severity by one or two groups (Fig. 2). Of those, 60.3% were male with a mean age of 57.2 years (*SD* = 16.8). The most frequently reported pre-existing comorbidity (mean 2.0 (SD = 2.0) was cardiovascular disease, which was present in 52.1% of patients. In contrast, patients whose PCS score improved over time (14.1%, 63.5% male) were younger (mean age 49.9 years (*SD* = 14.6)) and had fewer comorbidities (mean 1.5 (SD = 1.7) (Additional information in Table S9). At the 12MFU, the most symptom complexes were fatigue, exercise intolerance (representing dyspnea) and neurological ailments (indicating cognitive impairments). These symptom complexes had similar relative frequencies as at the 3MFU for both PCS scores with and without PROMs (Table S10).

### Functional aspects

At the 3MFU, 28.9% of patients (*n* = 160) exhibited social deprivations, while 41.9% (*n* = 236) had functional deficiencies. A group comparison between the PCS score and the selected PROMIS instruments revealed that only a few patients reported impairments without having a PCS according to the PCS score. Specifically, 6.7% (*n* = 8) of patients in the “no PCS” group had functional deficiencies according to PROMIS-29, and 1.7% (*n* = 2) reported social deprivation. Most of the patients’ impairments can be attributed to pre-existing comorbidities, primarily cardiovascular diseases (Table S11). For both instruments, the prevalence of impairments increased with PCS severity (Table 1). Correlation analysis also revealed a positive association between higher PCS score values and greater functional deficiencies (*p* < 0.001*, *rho* =−0.593) and social deprivation (*p* < 0.001*, *rho* = 0.635) (Table S8).

### Sensitivity analysis

Not considering missing data in symptom complexes may affect the results. We assessed the potential impact of missing data in a symptom complex by assigning 0 points in the PCS score. Our analysis revealed no major differences in the overall results compared to the main analyses (Table S12).

## Discussion

Post-COVID is a major public health concern [31] affecting both the individual’s QoL and society as a whole [15]. Applying the PCS score by Bahmer et al. in the cross-sectoral SUEP cohort of mostly hospitalized patients, our study showed differences in sex and QoL between PCS severity groups and significant correlations between PCS score and BMI, severity of acute infection, number of pre-existing comorbidities, and QoL. To adequately display the score in the SUEP cohort, we included PROMs in the score mapping. The calculation of the score identified 44.5% of patients with at least moderate PCS and 64.3% of patients with at least mild PCS outcome at 3MFU.

### Other existing definitions, severity classification, and scoring systems for Post-COVID

Ongoing endeavors are directed at characterizing and quantifying PCS in cohort studies. These methodologies predominantly emphasize the assessment of symptoms, the QoL, and ability to perform daily activities [13–16, 32]. In addition to Bahmer et al.'s PCS score, there are other definitions with a different focus or distribution.

Derived from the National Institute of Health’s *Researching COVID to Enhance Recovery* (RECOVER) in the United States, Thaweethai et al. sought to diagnose and identify PCS [15]. Their proposed symptom score attempts to diagnose PCS through a scoring system for 12 symptoms, selected for their specificity to PCS and differentiation from other causes of symptoms. Additionally, a cutoff for PCS positivity is established. A ORCHESTRA study by Gentilotti et al. [13] identified four clinical phenotypes in PCS: respiratory, chronic fatigue-like, neurosensory, chronic pain. PCS severity was assessed using the SF-36 questionnaire, which revealed diverse risk factors and impacts on QoL depending on the phenotype. Gentilotti et al. suggest that the PCS severity can be quantified based on the number of present clusters. Clustering approaches suggest that PCS may consists of multiple phenotypes and underlying pathomechanisms [13, 18].

In contrast, the PCS score by Bahmer et al. [14] focuses primarily on the severity and classification of PCS symptoms (see Methods for more information), but is not primarily designed for diagnosis per se. Although this score was developed based on three sites and a large sample size, the study did not reach the number of neither sites, participants, nor geographic heterogeneity of the RECOVER and ORCHESTRA studies. However, it has the advantage of being developed in a relatively large population-based sample and being designed as a potential marker of progression and possible outcome in clinical trials. We introduced this score in the SUEP cohort to harmonize the definitions and research activities within the NAPKON consortium and to test the transferability in another scenario. We have successfully demonstrated the clinically meaningful applicability of the PCS score in our study and contributed to the confidence of the score as a severity and classification tool.

### Selection of data items for score mapping and clinical applicability

In the POP study, Bahmer et al. did not initially include PROMs in the score development, arguing that “Asking the questions necessary to calculate the PCS score should take no longer than a few minutes and is therefore easy to implement in clinical practice.” [14]. However, transferring the score to other studies introduces new complexities that are closely connected to the type of symptom assessment used in each study. The use of PROMs has relevant drawbacks, including limited availability, constrained clinical applicability due to complexity, and practical barriers related to infrastructure and staff burden [33]. Nevertheless, PROMs also offer advantages such as confidence through validation, enhancing reliability compared to unstructured symptom inquiries, and objective measurements of patient perceptions. In our study, emphasizing the use of PROMs is essential to mitigate the potential underrepresentation of some symptoms (e.g., fatigue without PROMs 8.3%, with PROMs 41.1%) [9, 13] due to differences in the assessment methods and a potentially inadequate questioning. As demonstrated by this heterogeneous symptom prevalence, we highlight the importance of a standardized approach to symptom assessment, although this may reveal heterogeneous data quality. Consequently, in deriving implications and recommendations for other cohorts, it is crucial to consider that the PCS score will only be perceptible if either all relevant questions are already asked in some form or non-directly asked questions can be extracted from other parts of the dataset (e.g., PROMs). An initial evaluation of the frequency distribution of individual symptom complexes is important to assess the plausibility of approaches and ascertain whether fatigue might not be the predominant symptom.

First insights into the applicability of the PCS score and the quantification of PCS were demonstrated in a PCS outpatient clinic [34]. As the PCS score requires few resources (only one interview), it could be used at all levels of patient care and can be easily implemented in a trial if it is integrated from the beginning.

### Clinical relevance, quality of life and functional assessments

Despite the varying methodologies adopted across different cohorts, there exists a consensus among most researchers and clinicians regarding the significance of evaluating PCS in conjunction with QoL and functional capacities [3]. They utilize diverse QoL measurements as correlates to arrive at an applicable and clinically meaningful PCS definition (e.g., PCS score: EQ-5D-5L, Gentilotti et al. definition: SF-36) [13, 14]. When applying the PCS score [14] to the SUEP cohort, we observed comparable correlations between QoL assessed by EQ-5D-5L, functional deficits, social participation, and the PCS severity.

### Risk factors of PCS

Current research identifies female sex, increased age, pre-existing comorbidities, acute severity, and obesity as the most important determinants for PCS [35]. Our results align with this overall picture, as we identified inter-class differences for sex and pre-existing respiratory diseases. The correlation analysis revealed that higher BMI and a greater number of comorbidities were associated with more severe PCS. Although the role of psychiatric comorbidities was a major finding in the POP study, our investigation only revealed a tendency towards group differences that did not reach statistical significance. This observation may be attributed to disparities in the composition of the study population and the utilization of a smaller sample size. Although Bahmer et al. used other indicators of acute disease severity, such as number of symptoms, in a retrospective survey [14], we can confirm the correlation between acute severity based on the WHO progression scale, and higher PCS scores in the SUEP cohort. Especially the severity of the acute infection is a crucial factor in addressing PCS through either preventive intervention (e.g., vaccination), risk stratification or derived therapeutic decisions (e.g., medication for high-risk patient groups). Vaccination is a key protective factor for acute severity [36, 37], but its effect on PCS varies across studies [38]. Out study contributes to the ongoing debate by showing no differences in vaccination between PCS groups. However, this work did not investigate the effects of the type and timing of vaccinations in depth.

### Prevalence estimates and differentiation from pre-existing conditions

Given the heterogeneous definitions and population characteristics, the prevalence of PCS varies accordingly. Current literature mostly suggests a prevalence of PCS of 10–20% [3, 39], but studies describe a considerably range and risk of bias for prevalence estimates from 2–51% depending on the population studied, the hospitalization status at acute infection, and the PCS (severity) definition [9, 15, 35]. Bahmer et al. described the prevalence of “severe” PCS in the population-based cohort as 13–20% [14]. In the SUEP cohort, only 7.8% of patients had a severe PCS outcome according to the score. However, we identified at least moderate PCS in 44.5% and at least mild PCS in 64.3% of patients at 3MFU. Overall, the aggregated numbers appear to be rather high but still are in line with the literature, depending on the stratification and sensitivity. The observed discrepancies may be due to both the hospitalization rate (SUEP cohort: 83.8%) and differences in study methodology and design [35, 40]. Specifically, the separation of “none “ and “mild “ PCS groups significantly shifts in prevalence. The selection of an appropriate threshold is related to the discussion of the clinical relevance and treatment requirements of individual symptoms and their effects on QoL and functional capacity.

The existing research on PCS is challenged to address several pre-existing conditions [35]. It is crucial to distinguish PCS from comorbidities and psychiatric disorders, as functional impairments can often be attributed to these conditions. A study that corrected for symptoms prior to COVID-19 infection identified approximately one in eight individuals in the general population as experiencing PCS symptoms [12]. However, the baseline value, which indicated whether functional impairments were pre-existing, is often unavailable. Therefore, data analysis requires adjustments to isolate the effects of comorbidities. We hypothesize that many studies do not sufficiently adjust for pre-existing conditions. Apart from checking the duration of symptoms according to the WHO definition, no further adjustment for comorbidities was performed in our analysis, but these complexities should be addressed in the aftermath. Additionally, there may be other potential biases or explanations for the described symptoms, such as the Post-Intensive Care syndrome [41]. In summary, it is possible that we have overestimated the prevalence of PCS in the SUEP cohort.

## Strengths and limitations

The symptom mapping and methodology were discussed by a large interdisciplinary group of researchers. Furthermore, our analysis is based on a multi-center, prospective cohort study with in-depth assessments at 3 and 12 months after the acute infection. It is important to note that there are some limitations to our study, including missing baseline values for PROMs and the fact that the type of symptom assessment used in the SUEP cohort was not specifically designed for the targeted score. Approximately only 40% of the patients recruited in the SUEP were included in our analysis due to missing data at the 3MFU. An updated data export could potentially allow the analysis of more patients. The results indicate a low prevalence of high PCS among elderly patients (aged 65 years or older). Reasons for this may be that the respective patient group could not participate in the study or died before 3MFU, potentially limiting the generalizability of our results. Missing data were not handled using multiple imputation procedures. However, sensitivity analyses confirmed that there were no relevant differences between a complete case analysis and treating a missing symptom cluster as not present. This work focuses on the initial implementation of the PCS score in the SUEP cohort. Multivariable analyses were not aimed at in this study. Further analyses are needed to investigate the implications of risk factors and long-term outcomes of COVID-19.

## Conclusion

In conclusion, this study emphasizes the importance of standardized definitions and further investigation of the clinical presentation of PCS. The study confirms the applicability of the PCS score across health care settings for severity stratification and highlights its association with severity in acute infection. Furthermore, we demonstrated that the score generates clinically meaningful correlates of PCS and reflects patients ‘ functional status and quality of life. Hence, the score appears to be adequate to quantify the impact of symptoms and PCS severity. However, the high prevalence and score thresholds require further discussion. These findings are crucial for designing targeted studies on PCS pathogenesis, selecting high-risk patients for inclusion in clinical trials and clinical decision-making. Additionally, considering the impact of functional activity on social participation and work ability is critical for a comprehensive assessment of PCS.

## Materials/code availability

The code for PCS calculation will be made available in the epicodr^31^, a R-package developed by the Epidemiology Core Unit, an infrastructure of the NUM Clinical Epidemiology and Study Platform (NUKLEUS). The data are accessible through the NAPKON Use and Access procedure (https://napkon.de/use-and-access/).

## Supplementary Information

Below is the link to the electronic supplementary material.

Supplementary file1 (PDF 1127 KB)

## Data Availability

No datasets were generated or analysed during the current study.
