# Supplementary material for: Definition of the Post-COVID syndrome using a symptom-based Post-COVID score in a prospective, multi-center, cross-sectoral cohort of the German National Pandemic Cohort Network (NAPKON)
Source: Infection. 2024 Apr 8;52(5):1813–29. doi: 10.1007/s15010-024-02226-9 (PMC11499320; doi:10.1007/s15010-024-02226-9)
Supplement: Supplementary file 1 — Supplementary file1 (PDF 1127 KB) [file 15010_2024_2226_MOESM1_ESM.pdf]

Journal: Infection

Supplementary material to

Definition of the Post-COVID syndrome using a symptom-based Post-COVID score in a prospective, multi-center, cross-sectoral cohort of the German National Pandemic Cohort Network (NAPKON)

Katharina S. Appel[1,2*], Carolin Nürnberger[3,4*], Thomas Bahmer[5,6], Christian Förster[7], Maria Cristina Polidori[8,9], Mirjam Kohls[3], Tanja Kraus[7], Nora Hettich-Damm[10], Julia Petersen[10], Sabine Blaschke[11], Isabel Bröhl[2], Jana Butzmann[12], Hiwa Dashti[13], Jürgen Deckert[14], Michael Dreher[15], Karin Fiedler[1,2], Carsten Finke[16], Ramsia Geisler[1,2], Frank Hanses[17], Sina M. Hopff[18], Björn-Erik O. Jensen[19], Margarethe Konik[20], Kristin Lehnert[21,22], Susana M. Nunes de Miranda[2], Lazar Mitrov[18], Olga Miljukov[3,4], Jens-Peter Reese[3,4], Gernot Rohde[23], Margarete Scherer[1], Kristin Tausche[24], Johannes J. Tebbe[25], Jörg Janne Vehreschild[1,2,26], Florian Voit[27], Patricia Wagner[2], Martin Weigl[28], Christina Lemhöfer[29] on behalf of the NAPKON Study Group

*Contributed equally

1 Goethe University Frankfurt, University Hospital, Center for Internal Medicine, Medical Department 2 (Hematology/Oncology and Infectious Diseases), Frankfurt, Germany
2 University of Cologne, Faculty of Medicine and University Hospital Cologne, Department I of Internal Medicine, Cologne, Germany
3 University of Würzburg, Institute for Clinical Epidemiology and Biometry, Würzburg, Germany
4 University Hospital Würzburg, Institute for medical Data Science, Würzburg, Germany
5 Internal Medicine Department I, University Hospital Schleswig-Holstein Campus Kiel, Kiel, Germany
6 Airway Research Center North (ARCN), German Center for Lung Research (DZL), Grosshansdorf, Germany
7 Institute of General Practice and Interprofessional Care, University Hospital Tübingen, Tübingen, Germany
8 Department II of Internal Medicine and Center for Molecular Medicine Cologne, University of Cologne, Faculty of Medicine and University Hospital Cologne, Cologne, Germany
9 CECAD, University of Cologne, Faculty of Medicine and University Hospital Cologne, Cologne, Germany
10 Department of Psychosomatic Medicine and Psychotherapy, University Medical Center Mainz, Mainz, Germany
11 Emergency Department, University Medical Center Goettingen, Göttingen, Germany
12 Institute of Medical Microbiology and Hospital Hygiene, University Hospital Magdeburg, Medical Faculty, Otto-von-Guericke University Magdeburg, Magdeburg, Germany
13 Practice for general medicine Dashti, Eberswalde, Germany
14 University Hospital Würzburg, Center of Mental Health, Department of Psychiatry, Psychosomatics and Psychotherapy, Würzburg, Germany
15 Department of Pneumology and Intensive Care Medicine, University Hospital RWTH Aachen, Aachen, Germany
16 Department of Neurology, Charité Berlin, Berlin, Germany
17 Emergency Department and Department for Infection Control an Infectious Diseases, University Hospital Regensburg, Regensburg, Germany
18 University of Cologne, Faculty of Medicine and University Hospital Cologne, Department I of Internal Medicine, Center for Integrated Oncology Aachen Bonn Cologne Duesseldorf, Cologne, Germany
19 Department of Gastroenterology, Hepatology and Infectious Diseases, Düsseldorf University Hospital, Medical Faculty, Heinrich Heine University, Düsseldorf, Germany
20 Department of Infectious Diseases, West German Centre of Infectious Diseases, University Medicine Essen, University Duisburg-Essen, Essen, Germany
21 DZHK (German Center for Cardiovascular Research), University Medicine Greifswald, Greifswald, Germany
22 Department of Internal Medicine B, University Medicine Greifswald, Greifswald, Germany
23 Goethe University Frankfurt, University Hospital, Medical Clinic I, Department of Respiratory Medicine, Frankfurt/Main, Germany
24 Department of Internal Medicine I, University Hospital Carl Gustav Carus TU Dresden, Dresden, Germany
25 Klinikum Lippe, Department of Gastroenterology and Infectious Diseases, Lippe, Germany
26 German Centre for Infection Research (DZIF), partner site Bonn-Cologne, Cologne, Germany
27 Department of Internal Medicine II, University hospital rechts der Isar, Technical University of Munich, School of Medicine, Munich, Germany
28 Department of Orthopaedics and Trauma Surgery, Musculoskeletal University Center Munich (MUM), University Hospital, LMU Munich, Munich, Germany
29 Institute of Physical and Rehabilitation Medicine, Jena University Hospital/Friedrich-Schiller-University Jena, Jena, Germany

Corresponding author

Katharina S. Appel
Goethe University Frankfurt, University Hospital, Center for Internal Medicine, Medical Department 2 (Hematology/Oncology and Infectious Diseases), Frankfurt, Germany
Email: appel@med.uni-frankfurt.de

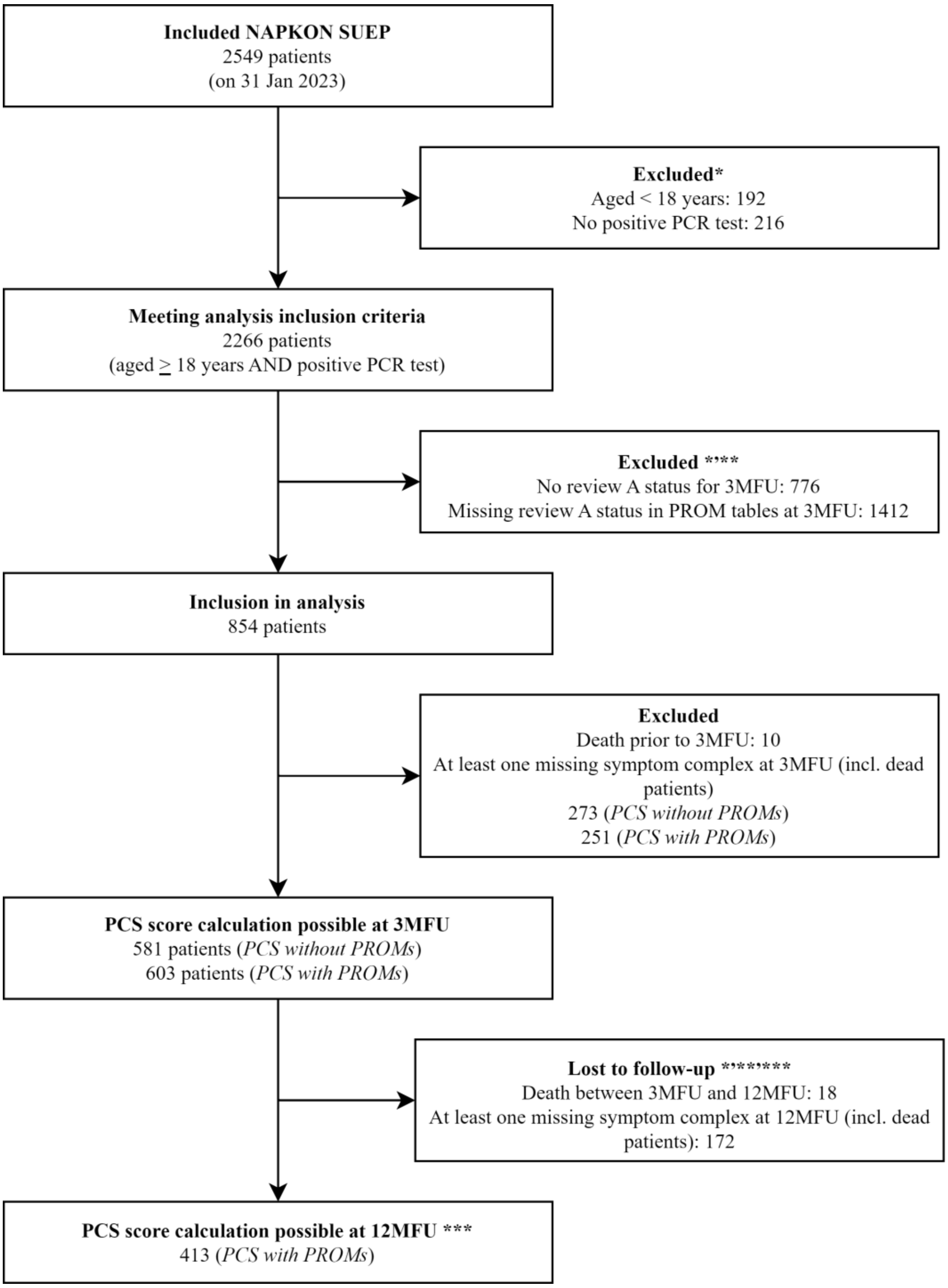

Figure S1: Study Flow Chart.

* at lest one criteria was not present; ** review A status means that a monitoring has been performed at the study sites, which recruited the patient and entered study data; this is a standard data export criterion for NAPKON data; *** PCS at 12MFU was only calculated for patients with PCS including PROMs at 3MFU

File S1: STROBE Statement—Checklist of items that should be included in reports of cohort studies.

	Item No	Recommendation	Page No
**Title and abstract**	1	(*a*) Indicate the study's design with a commonly used term in the title or the abstract	1
		(*b*) Provide in the abstract an informative and balanced summary of what was done and what was found	2
**Introduction**			
Background/rationale	2	Explain the scientific background and rationale for the investigation being reported	3
Objectives	3	State specific objectives, including any prespecified hypotheses	3
**Methods**			
Study design	4	Present key elements of study design early in the paper	4
Setting	5	Describe the setting, locations, and relevant dates, including periods of recruitment, exposure, follow-up, and data collection	4
Participants	6	(*a*) Give the eligibility criteria, and the sources and methods of selection of participants. Describe methods of follow-up	4
		(*b*) For matched studies, give matching criteria and number of exposed and unexposed	/
Variables	7	Clearly define all outcomes, exposures, predictors, potential confounders, and effect modifiers. Give diagnostic criteria, if applicable	4-5
Data sources/ measurement	8*	For each variable of interest, give sources of data and details of methods of assessment (measurement). Describe comparability of assessment methods if there is more than one group	4-5
Bias	9	Describe any efforts to address potential sources of bias	9
Study size	10	Explain how the study size was arrived at	4-5
Quantitative variables	11	Explain how quantitative variables were handled in the analyses. If applicable, describe which groupings were chosen and why	5
Statistical methods	12	(*a*) Describe all statistical methods, including those used to control for confounding	5
		(*b*) Describe any methods used to examine subgroups and interactions	5
		(*c*) Explain how missing data were addressed	5
		(*d*) If applicable, explain how loss to follow-up was addressed	4
		(*e*) Describe any sensitivity analyses	6

File S1: continued

**Results**			
Participants	13*	(a) Report numbers of individuals at each stage of study—eg numbers potentially eligible, examined for eligibility, confirmed eligible, included in the study, completing follow-up, and analysed	5
		(b) Give reasons for non-participation at each stage	5
		(c) Consider use of a flow diagram	/
Descriptive data	14*	(a) Give characteristics of study participants (eg demographic, clinical, social) and information on exposures and potential confounders	5
		(b) Indicate number of participants with missing data for each variable of interest	14-16
		(c) Summarise follow-up time (eg, average and total amount)	/
Outcome data	15*	Report numbers of outcome events or summary measures over time	5-6
Main results	16	(*a*) Give unadjusted estimates and, if applicable, confounder-adjusted estimates and their precision (eg, 95% confidence interval). Make clear which confounders were adjusted for and why they were included	14-15
		(*b*) Report category boundaries when continuous variables were categorized	4-5
		(*c*) If relevant, consider translating estimates of relative risk into absolute risk for a meaningful time period	
Other analyses	17	Report other analyses done—eg analyses of subgroups and interactions, and sensitivity analyses	6
**Discussion**			
Key results	18	Summarise key results with reference to study objectives	7
Limitations	19	Discuss limitations of the study, taking into account sources of potential bias or imprecision. Discuss both direction and magnitude of any potential bias	9
Interpretation	20	Give a cautious overall interpretation of results considering objectives, limitations, multiplicity of analyses, results from similar studies, and other relevant evidence	7-9
Generalisability	21	Discuss the generalisability (external validity) of the study results	9
**Other information**			
Funding	22	Give the source of funding and the role of the funders for the present study and, if applicable, for the original study on which the present article is based	11

*Give information separately for exposed and unexposed groups.

Table S1: Variable selection for PCS score mapping.

No.	Complex PCS	Description PCS	Symptom	Query	Form	Variables
1	**Chemosensory deficits**	Smelling disturbance, impaired sense of taste	Taste disorders	Symptoms	fv3_3	gec_sy_ne_taste
			Odor disorders	Symptoms	fv3_3	gec_sy_ne_smell
2	**Fatigue**	Fatigue	Fatigue (“Fatigue”)	Symptoms	esyoth	sy_oth_icd11name
			Chronic Fatigue Syndrome *(Persistent or frequent fatigue)*	PROM	prom	cfs
			Chalder Fatigue Scale	PROM	promext	cfs_seid_1 to cfs_seid_13
			PROMIS 29 – Fatigue	PROM	promext	pro_29_hi7, pro_29_an3, pro_29_fatexp41, pro_29_fatexp40
3	**Exercise intolerance**	Shortness of breath, reduced exercise capacity	Dyspnea	Symptoms	fv3_3	gec_sy_pd_dysp
			Dyspnea (screening question)	PROM	prom	dysp
			PROMIS Dyspnea	PROM	promext	pro_dysfl001 to pro_dysfl10
			Fatigue – diagnostic criteria *(Feeling sick after physical exertion)*	PROM	promext	cfs_seid_crit2
4	**Joint or muscle pain**	Muscle pain, joint pain	Joint pain	Symptoms	fv3_1	sy_arthr
			Muscle pain	Symptoms	fv3_1	sy_myalg
5	**Ear-nose-throat ailments**	Hoarseness, sore throat, running nose	Runny nose	Symptoms	fv3_2	sy_pd_rhin
			Nasal congestion	Symptoms	fv3_2	sy_pd_stuf
			Sneezing	Symptoms	fv3_2	sy_pd_sneez
			Sore throat	Symptoms	fv3_2	sy_pd_sore
6	**Coughing, wheezing**	Coughing, wheezing	Coughing	Symptoms	fv3_2	gec_sy_pd_cough
			Wheezing	Symptoms	fv3_2	sy_pd_wheez
7	**Chest pain**	Chest pain	Chest pain	Symptoms	fv3_1	sy_breastp
			Pain, location: chest	PROM	prom	pain_loc_chest
8	**Gastrointestinal ailments**	Stomach pain, diarrhea, vomiting, nausea	Stomach ache	Symptoms	fv3_2	gec_sy_gi_abdp
			Pain, location: abdomen	PROM	prom	pain_loc_abd
			Diarrhea	Symptoms	fv3_2	gec_sy_gi_diar
			Vomiting	Symptoms	fv3_2	gec_sy_gi_vom
			Nausea	Symptoms	fv3_2	gec_sy_gi_naus

Table S1: continued

No.	Complex PCS	Description PCS	Symptom	Query	Form	Variables
9	**Neurological ailments**	Confusion, vertigo, headache, motor deficits, sensory deficits, numbness, tremor, deficits of concentration, cognition or speech	Orientation disorder or confusion	Symptoms	fv3_3	gec_sy_ne_conf
			Cognitive impairments	Symptoms	Fv3_3	sy_ne_cogn
			Fatigue - diagnostic criteria (*Orthostatic intolerance - dizziness when standing up/walking*)	PROM	promext	cfs_seid_crit5
			Vertigo	Symptoms	fv3_1	sy_dizzi
			Headache	Symptoms	fv3_1	gec_sy_heada
			Pain, location: head	PROM	prom	pain_loc_head
			In the same area of the body: numbness (in case of pain)	PROM	promext	pain_dn2_6
			Fatigue - diagnostic criteria (*cognitive (concentration or word-finding) disorders*)	PROM	promext	cfs_seid_crit4
			PROMIS cognitive function	PROM	prom	pro_pc2r<br>pro_pc35r<br>pro_pc36r<br>pro_pc42r
10	**Dermatological ailments**	Hair loss, rash, itchiness	Change in the skin or mucous membranes	Symptoms	fv3_1	sy_skin
11	**Infection signs**	Chills, fever, general sickness/flulike symptoms	Feeling cold ("Chills")	Symptoms	esyoth	sy_oth_icd11name
			Fever	Symptoms	fv3_1	gec_sy_fever
			Anorexia	Symptoms	fv3_1	sy_appet
			Lymph node swelling	Symptoms	fv3_1	sy_lymph
			General sickness/flulike symptoms („Feeling ill")	Symptoms	esyoth	sy_oth_icd11name
12	**Sleep disturbance**	Insomnia, unrestful sleep	Sleep disturbance („*Sleep disturbance, not elsewhere classified*")	Symptoms	esyoth	sy_oth_icd11name
			PROMIS-29: Sleep disturbance	PROM	promext	pro_29_sleep109, pro_29_sleep116, pro_29_sleep20, pro_29_sleep44,

The complete data dictionary is available in German **here**.

Table S2: Post hoc test results for sex (male vs. female), smoker (yes or former vs. no), hospitalization (yes vs. no), administration to ICU (never vs. at least once), Vaccination against SARS-CoV-2 prior to study inclusion (yes vs. no).

	**p value**					
	PCC severity					
	None vs. mild	None vs. moderate	None vs. severe	Mild vs. moderate	Mild vs. severe	Moderate vs. severe
**Age**	.625	.718	.293	.373	.175	.430
**Sex**	.192	.078	**<.001***	.972	**.039***	**.032***
**Smoker**	.655	.570	.760	>.999	>.999	>.999
**Hospitalization**	.738	**.004***	.164	.051	.314	>.999
**Admission to ICU**	.456	.753	.514	.697	.208	.353
**At least one vaccination against SARS-CoV-2 prior to study inclusion**	.132	.366	.156	.496	.816	.403
**All vaccinations against SARS-CoV-2 prior to study inclusion**	**.019***	**.013***	.929	.825	.110	.131
**PROMIS-29 Ability to participate in social roles and activities**	**.004***	**<.001***	**<.001***	**<.001***	**<.001***	.054
**PROMIS-29 Physical Function**	**<.001***	**<.001***	**<.001***	**.001***	**.001***	.356

Table S3: Post hoc test results for comparison between PCC severity groups of age, BMI, number of comorbidities, PROMIS-29 Ability to participate in social roles and activities, PROMIS-29 physical function, number of vaccinations against SARS-CoV-2, EQ-5D-5L index and VAS at 3 and 12MFU, adjusted for multiple testing using Bonferroni correction.

	p value	power
**Difference in mean age between PCC groups**		
None vs. mild	.731	
None vs. moderate	.281	
None vs. severe	>.999	
Mild vs. moderate	>.999	
Mild vs. severe	>.999	
Moderate vs. severe	>.999	
**Difference in mean BMI between PCC groups**		
None vs. mild	>.999	
None vs. moderate	.442	
None vs. severe	.448	
Mild vs. moderate	.397	
Mild vs. severe	.361	
Moderate vs. severe	>.999	
**Difference in mean number of comorbidities between PCC groups**		
None vs. mild	.095	
None vs. moderate	**.045***	.128
None vs. severe	.263	
Mild vs. moderate	>.999	
Mild vs. severe	>.999	
Moderate vs. severe	>.999	
**Difference in mean of PROMIS-29 Ability to participate in social roles and activities sum score**		
**None vs. mild**	**<.001***	-.312
**None vs. moderate**	**<.001***	-.673
**None vs. severe**	**<.001***	-.737
**Mild vs. moderate**	**<.001***	-.350
**Mild vs. severe**	**<.001***	-.494
**Moderate vs. severe**	.431	
**Difference in mean of PROMIS-29 Physical function sum score**		
**None vs. mild**	**<.001***	-.332
**None vs. moderate**	**<.001***	-.616
**None vs. severe**	**<.001***	-.696
**Mild vs. moderate**	**<.001***	-.275
**Mild vs. severe**	**<.001***	-.437
**Moderate vs. severe**	.235	
**Difference in mean of number of vaccinations against SARS-CoV-2**		
**None vs. mild**	>.999	
**None vs. moderate**	>.999	
**None vs. severe**	>.999	
**Mild vs. moderate**	>.999	
**Mild vs. severe**	>.999	
**Moderate vs. severe**	>.999	
**Difference in mean of EQ-5D-5L Index at 3MFU**		
**None vs. mild**	**.006***	-.202
**None vs. moderate**	**<.001***	-.542
**None vs. severe**	**<.001***	-.597
**Mild vs. moderate**	**<.001***	-.323
**Mild vs. severe**	**<.001***	-.478
**Moderate vs. severe**	**.014***	-.146

Table S3: continued

	p value	power
**Difference in mean of EQ-5D-5L VAS at 3MFU**		
**None vs. mild**	**<.001***	-.280
**None vs. moderate**	**<.001***	-.557
**None vs. severe**	**<.001***	-.578
**Mild vs. moderate**	**<.001***	-.260
**Mild vs. severe**	**<.001***	-.385
**Moderate vs. severe**	.435	
**Difference in mean of EQ-5D-5L Index at 12MFU**		
**None vs. mild**	**<.001***	-.343
**None vs. moderate**	**<.001***	-.721
**None vs. severe**	**<.001***	-.719
**Mild vs. moderate**	**<.001***	-.358
**Mild vs. severe**	**<.001***	-.503
**Moderate vs. severe**	.327	
**Difference in mean of EQ-5D-5L VAS at 12MFU**		
**None vs. mild**	**.006***	-.233
**None vs. moderate**	**<.001***	-.619
**None vs. severe**	**<.001***	-.612
**Mild vs. moderate**	**<.001***	-.367
**Mild vs. severe**	**<.001***	-.484
**Moderate vs. severe**	.668	

Table S4: Post hoc test results for clinical severity (mild, moderate, severe) during acute infection.

	**p value**		
	Clinical severity during acute infection		
**PCC severity**	Mild vs. moderate	Mild vs. severe	Moderate vs severe
**None vs. mild**	.995	.245	.198
**None vs. moderate**	**.007***	**.014***	.613
**None vs. severe**	.147	.505	.806
**Mild vs. moderate**	**.036***	.402	.439
**Mild vs. severe**	.214	>.999	.255
**Moderate vs. severe**	>.999	.763	.530

Table S5: Post hoc test results for number of vaccinations against SARS-CoV-2 (0, 1, 2, 3 or more)

	**p value** [a]					
	Number of vaccinations against SARS-CoV-2 [b]					
**PCC severity**	0 vs. 1	0 vs. 2	0 vs. 3+	1 vs. 2	1 vs. 3+	2 vs. 3+
**None vs. mild**	.438	.991	.762	.552	.739	.906
**None vs. moderate**	.353	.064	.154	.635	.894	.806
**None vs. severe**	.606	.181	.325	.749	.984	.941
**Mild vs. moderate**	>.999	.192	.489	.245	.548	.654
**Mild vs. severe**	.267	.187	.231	>.999	>.999	>.999
**Moderate vs. severe**	.228	**.011***	.050	.503	.852	.782

Table S6: Group comparisons between PCC severity groups none, mild, moderate and severe stratified by sex and age groups.

	**Female**																	
	**< 65 years**									**>= 65 years**								
	**PCC Severity**									**PCC Severity**								
	**None**		**Mild**		**Moderate**		**Severe**		**p value [a]**	**None**		**Mild**		**Moderate**		**Severe**		**p value [a]**
**n (%)**	52	(28.7)	35	(19.3)	71	(39.2)	23	(12.7)		18	(32.1)	13	(23.2)	20	(35.7)	5	(9.0)	
**BMI [kg/m²],** *mean (SD)* [b,c]	26.7	(6.9)	26.2	(6.4)	29.3	(8.0)	29.9	(6.6)	.052	25.5	(6.3)	25.7	(7.1)	26.6	(4.4)	22.3	(3.1)	.377
**Smoker**, *n (%)* [b]																		
**Never**	39	(78.0)	21	(60.0)	46	(65.7)	15	(65.2)	.311	8	(47.1)	10	(76.9)	13	(65.0)	2	(40.0)	.309
**Yes or former**	11	(22.0)	14	(40.0)	24	(34.3)	8	(34.8)		9	(52.9)	3	(23.1)	7	(35.0)	3	(60.0)	
**Clinical Severity (following WHO criteria)** [b, f]																		
**Mild disease,** *n (%)*	20	(38.5)	12	(35.3)	12	(16.9)	2	(8.7)	.514	1	(5.6)	0		0		0		.162
**Moderate disease,** *n (%)*	28	(53.8)	19	(55.9)	54	(76.1)	19	(82.6)		17	(94.4)	10	(76.9)	16	(80.0)	5	(100.0)	
**Severe disease,** *n (%)*	4	(7.7)	3	(8.8)	5	(7.0)	2	(8.7)		0		3	(23.1)	4	(20.0)	0		
**Hospitalized**, *n (%)* [b]																		
**No**	20	(38.5)	12	(35.3)	12	(16.9)	2	(8.7)	**.035***	1	(5.6)	0		0		0		.643
**Yes**	32	(61.5)	22	(64.7)	59	(83.1)	21	(91.3)		17	(94.4)	13	(100.0)	20	(100.0)	5	(100.0)	
**Admission to intensive care unit (ICU)** [b]																		
**Never,** *n (%)*	28	(87.5)	16	(84.2)	47	(81.0)	18	(90.0)	.795	17	(100.0)	11	(84.6)	17	(85.0)	5	(100.0)	.323
**At least once,** *n (%)*	4	(12.5)	3	(15.8)	11	(19.0)	2	(10.0)		0		2	(15.4)	3	(15.0)	0		
	**Male**																	
	**< 65 years**									**>= 65 years**								
	**PCC Severity**									**PCC Severity**								
	**None**		**Mild**		**Moderate**		**Severe**		**p value [a]**	**None**		**Mild**		**Moderate**		**Severe**		**p value [a]**
**n (%)**	103	(40.1)	47	(18.3)	92	(35.8)	15	(5.8)		42	(39.3)	24	(22.4)	37	(34.6)	4	(3.7)	

**BMI [kg/m²]**, *mean (SD)* [b,c]	28.4	(5.4)	28.1	(5.9)	28.1	(4.3)	29.5	(4.5)	.573	26.8	(6.1)	27.9	(4.6)	28.2	(6.5)	27.3	(4.1)	.452

Table S6: continued

	**Male**																	
	**< 65 years**									**>= 65 years**								
	**PCC Severity**									**PCC Severity**								
	**None**		**Mild**		**Moderate**		**Severe**		**p value** [a]	**None**		**Mild**		**Moderate**		**Severe**		**p value** [a]
**Smoker**, *n (%)* [b]																		
**Never**	60	(59.4)	23	(48.9)	51	(56.0)	7	(46.7)	.587	19	(50.0)	14	(60.9)	16	(43.2)	3	(75.0)	.439
**Yes or former**	41	(40.6)	24	(51.1)	40	(44.0)	8	(53.3)		19	(50.0)	9	(39.1)	21	(56.8)	1	(25.0)	
**Clinical Severity (following WHO criteria)** [b, f]																		
**Mild disease,** *n (%)*	27	(26.2)	11	(23.4)	13	(14.4)	4	(26.7)	.327	2	(4.8)	2	(8.3)	2	(5.4)	0		.973
**Moderate disease,** *n (%)*	63	(61.2)	26	(55.3)	59	(65.6)	8	(53.3)		32	(76.2)	17	(70.9)	28	(75.7)	4	(100.0)	
**Severe disease,** *n (%)*	13	(12.6)	10	(21.3)	18	(20.0)	3	(20.0)		8	(19.0)	5	(20.8)	7	(18.9)	0		
**Hospitalized**, *n (%)* [b]																		
**No**	27	(26.2)	11	(23.4)	13	(14.4)	4	(26.7)	.196	2	(4.8)	2	(8.3)	2	(5.4)	0		.810
**Yes**	76	(73.8)	36	(76.6)	77	(85.6)	11	(73.3)		40	(95.2)	22	(91.7)	35	(94.6)	4	(100.0)	
**Admission to intensive care unit (ICU)** [b]																		
**Never,** *n (%)*	60	(83.3)	26	(74.3)	61	(80.3)	9	(81.8)	.733	30	(76.9)	17	(81.0)	30	(88.2)	4	(100.0)	.549
**At least once**, *n (%)*	12	(16.7)	9	(25.7)	15	(19.7)	2	(18.2)		9	(23.1)	4	(19.0)	4	(11.9)	0		

[a] group differences assessed by Chi2, Fishers- or Kruskal-Wallis test, as appropriate; normally significant values were Bonferroni-adjusted by multiplication with the size of the respective group characteristics (i.e. 6 or 18), if the unadjusted p value already exceeded 0.050, no adjustment was done

[b] all analyses were conducted for patients with available data (female missing data <65 years/>=65 years: BMI 16/6, smoker 3/1, admission to ICU 44/2, clinical severity 1/0, vaccination against Sars-COV-2 21/5; male missing data <65 years/>=65 years: BMI 21/8, smoker 3/5, hospitalization 1/0, admission to ICU 54/4, clinical severity 2/0, vaccination against SaSARS-CoV-2 32/15, PROMIS-29 Ability to participate in social roles and activities 74/35, PROMIS-29 Physical Function 71/34)

Table S7: Post hoc test results for smoker (yes or former vs. no), hospitalization (yes vs. no), administration to ICU (never vs. at least once) stratified by sex and age groups.

	**Female**											
	**p value**											
	**< 65 years**						**>= 65 years**					
	PCC severity						PCC severity					
	None vs. mild	None vs. moderate	None vs. severe	Mild vs. moderate	Mild vs. severe	Moderate vs. severe	None vs. mild	None vs. moderate	None vs. severe	Mild vs. moderate	Mild vs. severe	Moderate vs. severe
**Smoker**	.121	.209	.385	.720	.901	>.999	.201	.444	>.999	.701	.268	.358
**Hospitalization**	.945	**.013***	**.020***	.064	**.048***	.505	>.999	.474	>.999	-	-	-
**Administration to ICU**	>.999	.622	>.999	>.999	.661	.496	.179	.234	-	>.999	>.999	>.999

	**Male**											
	**p value**											
	**< 65 years**						**>= 65 years**					
	PCC severity						PCC severity					
	None vs. mild	None vs. moderate	None vs. severe	Mild vs. moderate	Mild vs. severe	Moderate vs. severe	None vs. mild	None vs. moderate	None vs. severe	Mild vs. moderate	Mild vs. severe	Moderate vs. severe
**Smoker**	.309	.745	.514	.540	>.999	.692	.575	.723	.608	.288	>.999	.321
**Hospitalization**	.869	.067	>.999	.283	>.999	.259	.618	>.999	>.999	.643	>.999	>.999
**Administration to ICU**	.398	.787	>.999	.644	>.999	>.999	>.999	.340	.564	.464	>.999	>.999

Table S8: Correlations with Post-COVID-Sum-Score.

	Total		Female				Male			
			< 65 years		>= 65 years		< 65 years		>= 65 years	
	**rho**	**p value**	**rho**	**p value**	**rho**	**p value**	**rho**	**p value**	**rho**	**p value**
**Age**	0.072	.077	-	-	-	-	-	-	-	-
**BMI**	0.121	**.004***	0.249	**.001***	0.023	.875	0.036	.584	0.196	.054
**Clinical Severity**	0.104	**.011***	0.226	**.002***	0.244	.070	0.106	.090	-0.017	.861
**Number of pre-existing comorbidities**	0.116	**.004***	0.092	.216	0.093	.494	0.170	**.006***	0.229	**.018***
**Number of vaccinations against SARS-CoV-2**	**0.036**	**.393**	**-0.042**	**.588**	**0.252**	**.069**	**0.009**	**.890**	**0.117**	**.254**
**Quality of Life (EQ-5D-5L) at 3MFU**										
**Index**	-0.558	**<.001***	-0.686	**<.001***	-0.277	.068	-0.551	**<.001***	-0.428	**<.001***
**VAS**	-0.531	**<.001***	-0.598	**<.001***	-0.278	.065	-0.554	**<.001***	-0.555	**<.001***
**Quality of Life (EQ-5D-5L) at 12MFU**										
**Index**	-0.691	**<.001***	-0.756	**<.001***	-0.244	.179	-0.698	**<.001***	-0.714	**<.001***
**VAS**	-0.594	**<.001***	-0.592	**<.001***	-0.416	**.018***	-0.602	**<.001***	-0.743	**<.001***
**PROMIS-29 Ability to participate in social roles and activities[1]**	-0.635	**<.001***	-0.668	**<.001***	-0.414	**.018***	-0.688	**<.001***	-0.520	**<.001***
**PROMIS-29 Physical function[1]**	-0,593	**<.001***	-0.646	**<.001***	-0.466	**.006***	-0.607	**<.001***	-0.520	**<.001***

[1] the lower the sum score, the higher the social or functional impairments

Table S9: Patient characteristics of patients with changed PCS from 3 to 12MFU.

	Worse PCS		No shift in PCS		Better PCS	
	**73**	**(12.1)**	**232**	**(38.5)**	**85**	**(14.1)**
**Sex**, *n (%)*						
**Women**	29	(39.7)	**96**	**(41.6)**	**31**	**(36.5)**
**Men**	44	(60.3)	**135**	(58.4)	54	(63.5)
**Age [years]**, *mean (SD)*	57.2	(16.8)	**53.6**	(15.5)	49.9	(14.6)
**Age groups**, *n (%)*						
**< 65 years**	45	(61.6)	**174**	(75.7)	69	(81.2)
**>= 65 years**	28	(38.4)	**56**	(24.3)	16	(18.8)
**Pre-existing comorbidities,** *n (%)*						
**Cardiovascular diseases**	38	(52.1)	**109**	(47.0)	29	(34.1)
**Diabetes**	15	(20.5)	**40**	(17.3)	9	(10.6)
**Cancer**	15	(20.5)	**29**	(12.6)	8	(9.4)
**Nephrological diseases**	12	(16.4)	**14**	(6.0)	10	(11.8)
**Allergies**	11	(15.3)	**39**	(16.9)	16	(18.8)
**Respiratory diseases**	7	(9.6)	**27**	(11.6)	15	(17.6)
**Gastrointestinal or hepatic diseases**	6	(8.2)	**18**	(7.8)	3	(3.5)
**Psychiatric diseases**	4	(5.5)	**11**	(4.7)	3	(3.5)
**Organ transplant**	3	(4.1)	**9**	(3.4)	5	(5.9)
**Neurological diseases**	2	(2.7)	**7**	(3.0)	3	(3.5)
**Rheumatologic or immunologic diseases**	1	(1.4)	**7**	(3.0)	2	(2.4)
**No. of pre-existing comorbidities,** *Mean (SD)*	2.0	(2.0)	**1.7**	(1.8)	1.5	(1.7)
**0, *n (%)***	19	(26.0)	**68**	(29.3)	31	(36.5)
**1-2, *n (%)***	30	(41.1)	**105**	(45.3)	36	(42.4)
**3-5, *n (%)***	18	(24.7)	**47**	(20.3)	14	(16.5)
**6 or more, *n (%)***	6	(8.2)	**12**	(5.2)	4	(4.7)
**No. of vaccinations per patient,** *Mean (SD)*	**1.4**	**(1.1)**	**1.5**	**(1.1)**	**1.5**	**(1.1)**
**0**, *n (%)* [b]	19	(26.8)	56	(25.3)	21	(27.3)
**1**, *n (%)* [b]	18	(25.4)	40	(18.1)	13	(16.9)
**2**, *n (%)* [b]	22	(31.0)	77	(34.8)	28	(36.4)
**3 or more**, *n (%)* [b]	12	(16.9)	48	(21.7)	15	(19.5)

Table S10: Symptom complexes with symptoms present for at least two months at 12MFU visit following the recommendations by Bahmer et al. (2022)

No.	Symptom complex	Self-reported sub-symptoms	Without PROMs		With PROMs	
			n	(%)	n	(%)
1	**Chemosensory deficits** [a, b]	Smelling disturbance, impaired sense of taste	45	(8.4)	45	(8.4)
2	**Fatigue** [b]	Fatigue	52	(9.2)	244	(42.6)
3	**Exercise intolerance**	Shortness of breath	96	(19.0)	205	(39.0)
4	**Joint or muscle pain** [a,b]	Muscle pain, joint pain	39	(7.4)	39	(7.4)
5	**Ear-Nose-Throat (ENT) ailments** [a,b]	Sneezing, sore throat, running nose, stuffy nose	16	(3.0)	16	(3.0)
6	**Coughing, wheezing** [a,b]	Coughing, wheezing	35	(6.5)	35	(6.5)
7	**Chest pain** [b]	Chest pain	11	(2.1)	63	(11.9)
8	**Gastrointestinal ailments** [b]	Stomach pain, diarrhea, vomiting, nausea	11	(2.0)	51	(9.0)
9	**Neurological ailments** [b]	Confusion, vertigo, headache, deficits of cognition	77	(14.0)	244	(43.3)
10	**Dermatological ailments** [a,b]	Skin or mucous membrane change	7	(1.3)	7	(1.3)
11	**Infection signs** [a,b]	Chills, fever, feeling ill, lymph node swelling, loss of appetite	14	(2.5)	14	(2.5)
12	**Sleep disturbance** [b]	Sleep disturbance, not elsewhere classified	0	-	78	(13.4)

[a] no adjustment with patient reported outcome measures (PROMs)

[b] percentages relate to number of patients with available data (missing data: chemosensory deficits 50, Fatigue without/with PROMs 17/12, exercise and intolerance without/with PROMs 81/59, joint or muscle pain 57, ENT ailments 48, coughing/ wheezing 43, chest pain without/with PROMs 63/57, gastrointestinal ailments without/with PROMs 21/20, neurological ailments without/with PROMs 36/22, dermatological ailments 53, infection signs 21, sleep disturbance without/with PROMs 2/2)

Table S11: Connection between social or functional impairments and pre-existing comorbidities for patients with no Post-COVID.

		PCC severity = "None"			
		PROMIS-29 Ability to participate in social roles and activities [a]		PROMIS-29 Physical Function [a]	
	Total	No impairments	Impairments	No impairments	Impairments
**Pre-existing comorbidities, *n (%)* [b]**	**854**	113 (13.2)	2 (0.23)	111 (13.0)	8 (0.94)
**Respiratory diseases**	10 (4.7)	2 (1.8)	0	2 (1.8)	0
**Cardiovascular diseases**	94 (43.7)	42 (37.2)	2 (100.0)	40 (36.0)	6 (75.0)
**Neurological diseases**	7 (3.3)	2 (1.8)	0	2 (1.8)	0
**Psychiatric diseases**	7 (3.3)	2 (1.8)	0	2 (1.8)	0
**Gastrointestinal or hepatic diseases**	11 (5.1)	5 (4.4)	0	7 (6.3)	0
**Diabetes**	29 (13.5)	14 (12.4)	0	14 (12.6)	2 (25.0)
**Rheumatologic or immunologic diseases**	1 (0.5)	1 (0.9)	0	1 (0.9)	0
**Nephrological diseases**	19 (8.8)	8 (7.1)	1 (50.0)	8 (7.2)	2 (25.0)
**Allergies** [j]	29 (13.5)	20 (17.7)	0	19 (17.1)	1 (12.5)
**Cancer** [j]	35 (16.3)	13 (11.5)	1 (50.0)	13 (11.7)	2 (25.0)
**Organ transplant** [j]	14 (6.5)	7 (6.2)	0	7 (6.3)	1 (12.5)

[a] all analyses were conducted for patients with available data (missing data: PROMIS-29 ability to participate in social roles and activities 172, PROMIS-29 Physical Function 166)
[b] all analyses were conducted for patients with available data (missing data: psychiatric diseases 1, diabetes 3, allergies 6, cancer 4)

Table S12: Sensitivity analysis treating a missing in a symptom complex as 0 points: Group comparisons between PCC severity groups none, mild, moderate and severe.

	PCC-Score [a]				p value [c]	
	**0 (none)**	**>0 to ≤10.75 (mild)**	**>10.75 to ≤26.25 (moderate)**	**>26.25 (severe)**	**unadjusted**	**adjusted**
**n (%)**	318 (37.2)	173 (20.3)	301 (35.2)	62 (7.3)		
**Age [years]**, *Mean (SD)* [b,d]	52.6 (17.1)	55.3 (16.7)	54.7 (14.9)	53.4 (13.8)	.218	n.a
**Age groups**, *n (%)* [b,d]						
**< 65 years**	226 (71.1)	122 (70.9)	224 (74.7)	51 (82.3)	.251	n.a
**>= 65 years**	92 (28.9)	51 (29.1)	76 (25.3)	11 (17.7)		
**Sex** *n (%)* [b,d]						
**Women**,	119 (37.4)	65 (37.8)	127 (42.2)	35 (56.5)	**.034***	.204
**Men**	199 (62.6)	107 (62.2)	174 (57.8)	27 (43.5)		
**BMI [kg/m²]**, *mean (SD)* [b,d]	27.6 (5.9)	28.2 (7.7)	28.4 (6.0)	29.1 (6.0)	.078	n.a
**Smoker**, *n (%)* [b,d]						
**Never**	179 (60.9)	100 (58.5)	166 (55.9)	35 (56.5)	.659	n.a
**Yes or former**	115 (39.1)	71 (41.5)	131 (44.1)	27 (43.5)		
**Clinical Severity (following WHO criteria)** [b,d]						
**Mild disease (no hospitalization)**, *n (%)*	64 (20.3)	31 (18.0)	35 (11.7)	7 (11.3)	**.026***	.466
**Moderate disease (hospitalized, no oxygen or oxygen by mask or nasal prongs (< 15 l/min))**, *n (%)*	204 (64.6)	105 (61.0)	208 (69.8)	48 (77.4)		
**Severe disease (hospitalized, oxygen by NIV or high flow (> 15 l/min))**, *n (%)*	48 (15.2)	36 (20.9)	55 (18.5)	7 (11.3)		
**Hospitalized**, *n (%)* [b,d]						
**No**	64 (20.3)	31 (18.0)	35 (11.7)	7 (11.3)	**.021***	.126
**Yes**	252 (79.7)	141 (82.0)	263 (88.3)	55 (88.7)		
**Admission to intensive care unit (ICU)** [b,d]						
**Never**, *n (%)*	202 (82.1)	104 (76.5)	209 (80.7)	46 (86.8)	.369	n.a
**At least once**, *n (%)*	44 (17.9)	32 (23.5)	50 (19.3)	7 (13.2)		
**Vaccination against SARS-CoV-2 prior to study inclusion** [b,e]						
**No**, *n (%)*	159 (57.8)	106 (67.5)	170 (60.7)	35 (60.3)	.262	n.a
**Yes**, *n (%)*	116 (42.2)	51 (32.5)	110 (39.3)	23 (39.7)		
**No. of pre-existing comorbidities**, *Mean (SD)* [b,d]	1.6 (1.8)	2.0 (2.1)	2.0 (2.0)	2.3 (2.3)	.053	n.a
**0,** *n (%)*	106 (33.3)	56 (32.4)	83 (27.6)	15 (24.2)	.276	n.a
**1 or more,** *n (%)*	212 (66.7)	117 (67.6)	218 (72.4)	47 (75.8)		

Table S12: continued

	PCC-Score [a]				p value [c]	
	**0 (none)**	**>0 to ≤10.75 (mild)**	**>10.75 to ≤26.25 (moderate)**	**>26.25 (severe)**	**unadjusted**	**adjusted**
**Pre-existing comorbidities** *n (%)* [b,f]						
**Cardiovascular diseases**	140 (44.0)	84 (48.6)	139 (46.2)	26 (41.9)	.729	n.a
**Diabetes**	44 (14.0)	40 (23.3)	42 (14.0)	11 (17.7)	**.035***	.210
**Cancer**	47 (15.0)	20 (11.6)	53 (17.7)	15 (24.6)	.083	n.a
**Allergies**	43 (13.8)	26 (15.0)	45 (15.1)	9 (14.5)	.969	n.a
**Respiratory diseases**	24 (7.6)	21 (12.1)	52 (17.3)	14 (22.6)	**<.001***	**.002***
**Nephrological diseases**	28 (8.8)	19 (11.0)	32 (10.6)	10 (16.1)	.356	n.a
**Gastrointestinal or hepatic diseases**	21 (6.6)	11 (6.4)	21 (7.0)	6 (9.7)	.794	n.a
**Organ transplant**	20 (6.3)	9 (5.2)	14 (4.7)	4 (6.5)	.773	n.a
**Psychiatric diseases**	11 (3.5)	5 (2.9)	26 (8.7)	5 (8.3)	**.009***	.055
**Neurological diseases**	12 (3.8)	8 (4.6)	11 (3.7)	7 (11.3)	.087	n.a
**Rheumatologic or immunologic diseases**	5 (1.6)	4 (2.3)	17 (5.7)	2 (3.2)	**.033***	.200
**PROMIS-29 Ability to participate in social roles and activities** [b,d]	18.9 (2.0)	16.9 (3.8)	13.1 (4.3)	11.3 (3.9)	**<.001***	**<.001***
**No social impairments**, *n (%)*	144 (98.6)	104 (89.7)	128 (53.6)	17 (32.7)	**<.001***	**<.001***
**Social impairments**, *n (%)*	2 (1.4)	12 (10.3)	111 (46.4)	35 (67.3)		
**PROMIS-29 Physical function** [b,d]	19.4 (1.6)	17.6 (3.5)	15.2 (4.2)	13.7 (4.2)	**<.001***	**<.001***
**No physical impairments**, *n (%)*	140 (44.0)	81 (46.8)	93 (30.9)	13 (21.0)	**<.001***	**<.001***
**Physical impairments**, *n (%)*	12 (3.8)	38 (22.0)	148 (49.2)	38 (61.3)		

[a] PCC-Groups including PROMs; missing values: 22; missings in symptom complexes were ignored (represented 0 points for the complex)

[b] all analyses were conducted for patients with available data (missing data by PCC-group:, age/age groups 2, sex: 1, BMI 82, smoker 30, clinical severity 6, hospitalization 6, admission to ICU 127, vaccination against SARS-CoV-2 prior to study inclusion 84, PROMIS-29 Ability to participate in social roles and activities 301, PROMIS-29 Physical function 291, diabetes 6, cancer 7, allergies 9, respiratory diseases 1, psychiatric diseases 5)

[c] group differences assessed by Chi2, Fishers- or Kruskal-Wallis test, as appropriate; normally significant values were Bonferroni-adjusted by multiplication with the size of the respective group characteristics (i.e. 6 or 18), n.a: not applicable because the unadjusted p value already exceeded 0.050

[d] for post-hoc test results see Figure 3 (age), Figure 4 (BMI), Table 4 (age groups (< or >= 65 years), sex, smoker, hospitalization, administration to ICU), Table 5 (clinical severity)

[e] "No" includes all patients without any SARS-CoV-2 vaccination

[f] pre-existing comorbidities were either diagnosed or not diagnosed

Supplementary text 1: Version numbers of PROMs

In the SUEP, PROMIS were assessed in the following versions: PROMIS-29 Fatigue 4a – Adult v1.0, PROMIS-29 Dyspnea Functional Limitations 10a – Adult v1.0, PROMIS Cognitive Function 4a – Adult v2.0, PROMIS-29 Sleep Disturbance 4a.
